# Atrial Fibrillation, thromboembolic risk, and the potential role of the natriuretic peptides, a focus on BNP and NT-proBNP – A narrative review

**DOI:** 10.1016/j.ijcha.2022.101132

**Published:** 2022-10-10

**Authors:** Brian Kerr, Lisa Brandon

**Affiliations:** Department of Cardiology, St James Hospital, James Street, Dublin 8, Ireland

**Keywords:** Atrial fibrillation, Natriuretic peptides, BNP, NT-proBNP, Atrial cardiomyopathy, ACM, Atrial cardiomyopathy, AF, Atrial fibrillation, ARISTOTLE trial, Apixaban For Reduction In Stroke And Other Thromboembolic Events In Atrial Fibrillation Trial, ASSERT trial, Atrial Fibrillation Evaluation In Pacemaker Patient’s Trial, ASSERT-II trial, Asymptomatic Atrial Fibrillation and Stroke Evaluation in Pacemaker Patients and the Atrial Fibrillation Reduction Atrial Pacing Trial, AUC, Area Under The Curve, BNP, Brain natriuretic peptide, CE, Cardioembolic, CHA_2_DS_2_-Vasc, Congestive Heart Failure, Hypertension, Age ≥ 75, Diabetes, Stroke/TIA/Thromboembolism, Vascular Disease, Age 65–74, CHARGE, Cohorts For Heart And Aging Research In Genomic Epidemiology, CI, Confidence Intervals, CNP, C-type natriuretic peptide, EHRAS, EHRA/ HRS/APHRS/SOLAECE, ESUS, Embolic Stroke of Unknown Source, IMPACT Trial, Implementation of An RCT To Improve Treatment With Oral Anticoagulants In Patients With Atrial Fibrillation, MR-proANP, Mid Regional Pro-Atrial Natriuretic Peptide, NP, Natriuretic peptide, NT-proBNP, N-Terminal Pro Brain Natriuretic Peptide, RE-LY study, The Randomized Evaluation of Long-Term Anticoagulation Therapy study, SE, Standard Error, TE, Thromboembolic event, TIA, Transient ischemic attack, TRENDS trial, A Prospective Study of the Clinical Significance of Atrial Arrhythmias Detected by Implanted Device Diagnostics

## Abstract

Atrial fibrillation (AF) is one of the most commonly encountered arrythmia in clinical practice. AF itself can be driven by genetic predisposition, ectopic electrical activity, and abnormal atrial tissue substrates. Often there is no single etiological mechanism, but rather a combination of factors that feed back to remodel and worsen tissue substrate, “AF begets AF”. The clinical consequences of AF can often include emboli, heart failure, and early mortality. The classical AF cardioembolic (CE) concept requires thrombus formation in the left atrial appendage, with subsequent embolization. The temporal dissociation between AF occurrence and CE events has thrown doubt on AF as the driver of this mechanism. Instead, there has been a resurgence of the “atrial cardiomyopathy” (ACM) concept. An ACM is proposed as a potential mechanism of embolic disease through promotion of prothrombotic mechanisms, with AF instead reflecting atrial disease severity. Regardless, AF has been implicated in 25% to 30% of cryptogenic strokes. Natriuretic peptide(NP)s have been shown to be elevated in AF, with higher levels of both NT-proBNP and BNP being predictive of incidental AF. NPs potentially reflect the atrial environment and could be used to identify an underlying ACM. Therefore, this narrative review examines this evidence and mechanisms that may underpin the role of NPs in identifying atrial dysfunction, with focus on both, BNP and NTproBNP. We explore their potential role in the prediction and screening for both, ACM and AF. Moreover, we compare both NPs directly to ascertain a superior biomarker.

## 1 Introduction

Atrial fibrillation (AF) is associated with an increased risk of morbidity such as stroke/transient-ischemic attack(TIA), heart failure and cognitive impairment[1], [2], and is also linked to increased all-cause mortality[2], [3]. Patients’ quality of life can be disrupted, while 10 %-40 % of patients are hospitalized due to their AF each year[4], [5], [6]. Stroke/TIA is the most dreaded consequence of AF, and can be largely mitigated against by the introduction of anticoagulation therapy.

## 2 Mechanism of atrial fibrillation

Over the years, we have learned that AF is driven by genetic predisposition, ectopic electrical activity, and abnormal atrial tissue substrate which then feeds back to remodel and worsen tissue substrate, thereby propagating the phrase, “AF begets AF”[7]. There is no single etiologic mechanism for AF. Rather, different mechanisms, sometimes singly, but usually in combination, play a role in the genesis and maintenance of AF. Broadly, this involves an electrical trigger facilitated by a vulnerable substrate[8]. The vulnerable substrate is usually the result of structural remodeling following an external stressor (e.g., hypertension, heart disease, ischemic etc.) and subsequently activates fibrotic, inflammatory and oxidative pathways. In contrast to electrophysiological remodeling, this structural remodeling occurs on a longer timescale, over months or even years[9].

The basis for maintaining AF is more complex and controversial. Currently-two theories exist; focal triggers and a re-entrant mechanism[10]. It’s likely that a combination of the two mechanisms occur in order to maintain AF[11]. The re-entrant mechanisms are debated but are likely the result of either rotors[12], which are functional re-entrant sources generating spiral waves, or endo-epicardial dissociation[13] that produce bi-directional conduction between two layers of atrial wall.

## 3 Thromboembolic risk in AF

The clinical consequences of AF are of paramount importance and include; emboli, heart failure, and early mortality[14]. Once detected, AF warrants anticoagulation when an individual’s CHADS_2_ or CHA_2_DS_2_-VASc is ≥ 2 in order to minimize embolic risk[15]. However, the detection of AF can be difficult in clinical practice, mostly due to the often silent nature of the rhythm [16]. This is important as 25 % to 30 % of patients presenting with strokes are diagnosed with incidental AF [16]. Moreover, AF is one of the most common underlying causes of cryptogenic stroke[17], with a prevalence of 25 % to 30 %[16].

The classical concept of stroke/TIA in AF revolves around the formation of a thrombus in the left atrial appendage, that then dislodges embolizing to the cerebral circulation causing an occlusion[18]. In reality, the mechanism of AF thrombogeneses is multifactorial which satisfies all aspects of Virchow’s triad; abnormal stasis of blood, endothelial dysfunction, and hypercoagulability[19]. For example, AF is associated with increased levels of prothrombotic factors (d-dimers, Von Willebrand Factors and Interleukin-6), left atrial enlargement and hypo contractility, as well as endothelial hypertrophy and fibrosis[10]. Nevertheless, it is also evident that AF itself may not be the sole cause of thromboembolism(TE) or stroke/TIA.

Initially AF was considered one of the most important underlying mechanisms in embolic stroke of unknown source (ESUS) patients, with 30 % of ESUS patients diagnosed with AF during long-term follow-up [17], [20], [21]. However, the ASSERT (Asymptomatic Atrial Fibrillation and Stroke Evaluation in Pacemaker Patients and the Atrial Fibrillation Reduction Atrial Pacing Trial) [22] and TRENDS (A Prospective Study of the Clinical Significance of Atrial Arrhythmias Detected by Implanted Device Diagnostics)[23] trials demonstrated a temporal dissociation between AF and embolic events. The casual association between AF and ESUS has been weakened further by the FIND-AF trial[24]. This trial demonstrated that AF detection during follow-up in ESUS patients is similar to other non-ESUS stroke patients. Meanwhile, in the ASSERT-II trial (Asymptomatic Atrial Fibrillation and Stroke Evaluation in Pacemaker Patients and the Atrial Fibrillation Reduction Atrial Pacing Trial)[25], the degree of subclinical atrial fibrillation lasting > 5 min was similar among older patients with and without a history of stroke. The Implementation of an RCT to IMprove Treatment with oral AntiCoagulanTs in patients with atrial fibrillation (IMPACT)trials[26] showed that AF events were identified within the 30 days before their stroke in only 8 % of individuals, with a first episode of AF detected post stroke in 16 % of stroke victims. In fact, it has been suggested that the hypercoagulable state itself could promote the development of AF[27]. Moreover, maintenance of sinus rhythm with anti-arrhythmic drugs in AF has not been shown to be beneficial to reduce stroke risk in comparison to patients who are rate controlled[28]. Although this is contradictory to the results of the Atrial fibrillation/Atrial flutter (ATHENA) trial, which confirmed that dronedarone reduced the incidence of hospitalization due to cardiovascular events or death in patients with paroxysmal/persistent AF compared with placebo, it’s likely this was the result of pleiotropic effects rather than the rhythm-control effects of dronedarone[29], [30].

Finally, current stroke prophylaxis strategies in AF include; anticoagulation and left atrial appendage occlusion devices[31]. These address only 2 of the mechanisms of Virchow triad. Currently, no proven therapies are directed at improving atrial dilation and hypo contractility. As a result of the above, the “atrial cardiomyopathy”(AF) concept has emerged as a potential mechanism of embolic disease in AF. Moreover, the atrial dysfunction thought to encompass an ACM itself can cause stroke through atrial hypo-contractility, generalized and local prothrombotic effects, and an inflammatory state [32]. Similar to AF, a link to cognitive decline is emerging[19]. Atrial cardiomyopathy is defined by the EHRAS (EHRA/HRS/APHRS/SOLAECE) as, “Any complex of structural, architectural, contractile or electrophysiological changes affecting the atria with the potential to produce clinically-relevant manifestations”[28]. It is then further divided into 4 EHRAS classes (cardiomyocyte dependent, fibroblast dependent, mixed fibroblast and cardiomyocyte, and non–collagen deposit related) according to their histopathological characterization[28]. Currently, no clinically universal definition exists for ACM.

## 4 Natriuretic peptides and evidence in AF

Baseline natriuretic peptide(NP)s have been shown to predict stroke, with *N*-Terminal Pro Brain Natriuretic Peptide (NT-proBNP) levels correlating to the degree of arteriosclerosis[33] and carotid plaque burden[34], both of which are associated with ischaemic stroke[35], [36]. They are also frequently and significantly increased after an acute ischemic stroke[37], [38], even when adjusted for age and sex[39]. Similarly, mid regional pro-atrial natriuretic peptide (MR-proANP) has been identified as a biomarker of CE(cardioembolic) stroke but not small vessel cerebral disease, with at least similar associations to both NT-proBNP and Brain Natriuretic Peptide (BNP)[40]. They also appear to remain significantly elevated for up to 72 h after the acute event[41] and could be used to differentiate a CE origin of ischemic stroke from other subtypes of ischemic stroke[42], [43]. They have also shown to outperform both troponin and d-dimers in this regard[44]. There are a few theories to explain this epiphenomenon between NPs and CE stroke. The first, and the most often cited, is the link between the elevated NPs and AF. However, NP release consequential of cerebral ischemia or other neurodegenerative processes, during and following a stroke[45] cannot be ruled out. Moreover, it is also important to interpret NPs in the context of renal failure. Chronic kidney disease, a risk factor for stroke, also down regulates neutral endopeptidase expression leading to a potential coincidental increase in NP levels in stroke[46], [47].

NPs have been shown to be elevated in AF[48], [49], with higher levels in patients with sustained AF than paroxysmal AF[48]. Atrial natriuretic peptide (ANP) is secreted predominantly by the atria, which could therefore theoretically more accurately identify AF. Indeed, ANP plasma concentrations are increased in patients presenting with AF[50]. Even so, its short half-life means its usefulness in clinical practice is questionable[51]. Nevertheless, MR-proANP, a stable fragment of the ANP precursor hormone, can be accurately and repeatedly measured, and has been correlated with incidental AF, with higher levels suggestive of an episode of AF[50]. However, an inverse relationship between ANP and long-standing AF also appears to exist. This likely reflects the degree of structural change, with lower levels of ANP seen in these patients[52]. C-type natriuretic peptide (CNP), another NP, in contrast to both ANP and BNP (which are secreted from cardiomyocytes), is an endothelial product synthesized in vascular beds including the heart, and is released in response to stimuli including shear stress and cytokines[53]. In experimental studies, CNP has demonstrated the ability to suppress cardiac fibroblast proliferation, collagen synthesis, and myocardial fibrosis, therefore it could be inferred that in AF, CNP could be elevated as a compensatory mechanism to reverse myocardial fibrogenesis seen in AF[53]. There is only emerging data regarding the possible link between CNP levels and AF [53], [54]. As a result of these findings, the wider knowledge base, and evidence for BNP/NT-proBNP in AF prediction[40], as well as the lack of direct comparison, and integration of the other aforementioned NPs into clinical practice[40], [55], BNP and NT-proBNP will be the main focus and reference for NPs in this review (Table 1).

**Table 1:** Selected studies demonstrating the degree of association of NT-proBNP and BNP with incidental AF.

Year	Author	Study type	NTproBNP HR (95 % CI)	BNP HR (95 % CI)
2009	Patton et al. (74)	Longitudinal study	4.0 (3.2–5.0)	NA
2004	Wang et al. (72)	Prospective	2.09 (1.21–3.62)	1.91 (1.13–3.25)
2010	Smith et al. (75)	Prospective	1.63 (1.29–2.06)	NA
2014	Sinner et al. (76)	Metanalysis	NA	1.66 (1.56–1.76)
2013	Patton et al. (73)	Prospective	2.2 (1.9–2.5)	NA

**Fig. 1:**
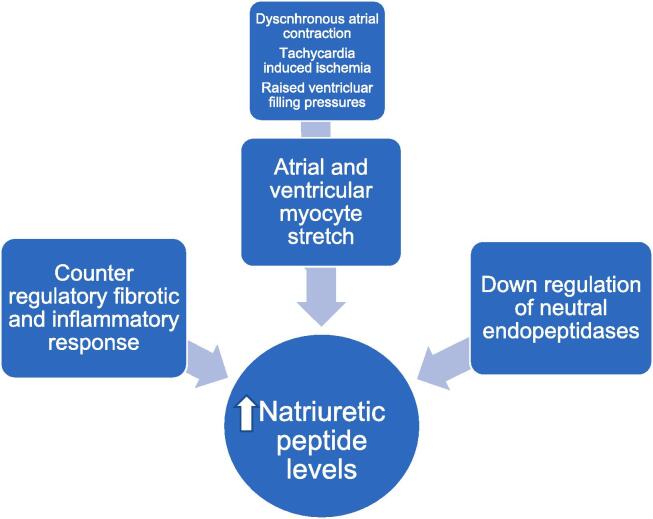
Potential mechanism(s) of NP release in AF. Abbreviations: AF = Atrial fibrillation, NP = Natriuretic peptide.

Abbreviations; AF = Atrial fibrillation, BNP = Brain natriuretic peptide, CI = Confidence interval, HR = Hazard ratio, NA = Not applicable, NT-proBNP = *N*-Terminal Pro Brain Natriuretic Peptide,

The increase of NT-proBNP in AF patients can result from both atrial and ventricular production(Fig. 1). During episodes of AF, both NPs are potentially released in small quantity from the atrium following dyssynchronous contraction of the atrial myocardium, producing a tethering effect on atrial myocardial fibers that may stimulate the secretion of BNP [56], [57]. Loss of atrial contraction and elevation of atrial pressure stretches the atrial wall leading to an unfavorable alternation of the left ventricular filling pattern, with subsequent ventricular production of natriuretic peptides[58]. It’s also thought that an increased ventricular rate during AF leads to oxygen mismatch, myocardial ischemia, further volume and pressure overload, and changes in microvascular blood flow, thus resulting in the ventricular production of natriuretic peptides[58]. It was also demonstrated that strict rate control yielded a prominent decrease in BNP values in AF patients[59].

It is also thought that a raised NT-proBNP may signal atrial dysfunction. NPs possess antifibrotic effects, with plasma NPs being found to correlate with established serum markers of collagen I turn-over (carboxyterminal telopeptide of collage type I and tissue inhibitor matrix metalloproteinase 1)[60]. Previously strong correlations between baseline BNP, atrial tissue M2 macrophage CD163+, procollagen expression, and subsequent atrial fibrosis in a cohort of patients with and without AF have been established[61]. As a result, NPs levels could proportionally reflect the counter-regulatory responses, but not the cause of proinflammatory and profibrotic mechanism(s) in atrial cardiomyopathies[62]. This may explain why increased NP levels are associated with an increased risk of stroke and death in patients with AF, a Randomized Evaluation of Long-term anticoagulation therapY (rE-LY) biomarker study[63] was the first to establish this connection. This study demonstrated that higher levels of NT-proBNP correlated with higher risk of TE events and cardiovascular mortality, even when adjusted for known risk factors. Following this, the larger Apixaban for the prevention of Stroke in subjects with atrial fibrillation(ARISTOTLE) biomarker sub study[48] verified the results of the rE-LY sub study. Similar to the rE-LY trial, the ARISTOTLE biomarker sub-study confirmed that the addition of NT-proBNP to the CHADS_2_ and CHA_2_DS_2_-VASc models resulted in significant improvements in their discrimination performance for both TE events and cardiovascular mortality (c statistic Aristotle: stroke 0.62 to 0.65 and cardiovascular death 0.59 to 0.69). Interestingly, one prospective trial in non-anticoagulated AF who underwent transesophageal echocardiography has suggested that BNP levels > 251.2 pg/mL are independent predictors for left atrial thrombi[64]. This could have implications regarding further investigation for left atrial appendage thrombus in patients presenting for cardioversion for AF, particularly in the first 48 h of rhythm onset. Finally, there is evidence to suggest that AF, downregulates neutral endopeptidase expression, thus leading to increased NP levels[65].

Cardiovascular risk factors such as age, hypertension and coronary disease etc. are associated with raised NPs, and can also promote ventricular dysfunction leading to atrial fibrillation/ACM [66], [67], [68], [69], [70]. Therefore, an alternative view could suggest a putative role of NP in causing AF. This seems dubious, particularly in the case if NT-proBNP, where it is unlikely to mediate any cardiovascular effect as it does not activate any NP receptors or independent NP receptors[51]. Moreover, in the case of nesiritide (recombinant human BNP), no reports of excess incident AF have emerged[71]. In fact, natriuretic peptides suppress both sympathetic traffic and renin– angiotensin–aldosterone activity. Overall, the actions of NPs appear cardioprotective and would mitigate against atrial remodeling[51].

Sub-clinical AF can often be difficult to detect. However, NPs could help predict the development of AF independent of heart failure, and other traditional risk factors[72], [73] (Table 1). A report from both the Cardiovascular Health Study[74] and the Malmö Diet and Cancer Study[75] found that higher NT-proBNP levels were significantly associated with incident AF events after adjustment for common risk factors. Additionally, the Framingham Heart Study(FHS)[72] showed that NT-proBNP could predict incident AF. BNP was also found to improve the predictive ability of the Cohorts for Heart and Aging Research in Genomic Epidemiology AF consortium (CHARGE-AF) risk score for AF[76].

## 5 The potential role of NPs in AF screening

Currently the European Society of Cardiology guidelines on AF management do not recommend the use of NPs for AF screening. Instead, they recommend opportunistic screening in over 65s via a pulse check, or an ECG rhythm strip, and regular checks of a pacemaker or implantable cardiac defibrillator for atrial high rates. In post stroke patients they recommend at least 72 h of continuous cardiac monitoring in order to identify AF [15].

However, given the evidence outlined previously, particularly in the peri-stroke/cryptogenic stroke population, NPs could aid in identifying patients who may benefit from an implantable loop recorder, or other means of prolonged monitoring (outside the scope of the European Society of Cardiology recommendation). Recent studies have further stratified the predictive value of NPs in detecting AF. For example, to date, NT-proBNP levels of < 95 pg/ml and < 125 pg/ml have been associated with a negative predictive value of between 86 % and 98 %[77], [78], [79], [80], [81]. Currently 1 % of total healthcare spending in the United Kingdom, and between 6.0 and 26.0 billion dollars in the Unites States for 2008 were as a direct result of AF[15]. Therefore, screening for AF using NPs could ring fence precious health care resources for at risk cohorts, while also reducing associated healthcare costs. Svennberg et al[77] previously reduced the need for AF screening by 35 % by using a NT-proBNP cut of level of < 125 pg/ml, and thus reduced the cost of ECG equipment and ECG interpretation[77]. Clinically, this could translate to earlier identification of patients with, or at risk of AF. This could facilitate earlier commencement of anticoagulation, atrial modifying therapies, and other lifestyle and risk factor modifications in those diagnosed, or risk of AF. The net outcome may result in a reduction in the burden of AF and its downstream effects, on both patients and healthcare systems. A universally defined NT-proBNP or BNP cut off level to optimize AF screening, depending on the clinical setting and population under evaluation, requires further investigation.

## 6 BNP or NT-proBNP for AF screening

Despite a multitude of studies demonstrating that both NPs can predict AF[79], [82], [83], little is known on whether one NP possesses superior ability over the other for AF prediction (Table 2). Moreover, data also indicates that both BNP and NT-proBNP are strongly correlated to each other: r = 0.89[84] and r = 0.87[85]. During episodes of AF, both NPs are potentially released in small quantity from the atrium as outlined above[56], [57], [86]. BNP and NT-proBNP are secreted in equimolar concentrations into the blood stream after the cleavage of their precursor pro-BNP[87], but BNP has a shorter half-life than NT-proBNP[88]. Theoretically, it would make sense for BNP to be a more specific marker of AF than NT-proBNP. Despite having a shorter half-life, higher BNP higher levels in circulation may reflect continuous secretion by cardiac myocytes due to atrial dysfunction, a known substrate for AF. Moreover, NT-proBNP has been shown to be more influenced than BNP by other cardiac and non-cardiac conditions, such as in renal dysfunction[87].

**Table 2:** Selected studies directly comparing predictive power of NT-proBNP and BNP.

Year	Author	Study type	NTproBNP AUC (95 % CI or SE)	BNP AUC (95 % CI or SE)
2012	Wachter et al. (93)	Prospective, observational	0.638 (0.531–0.744)	0.747 (0.663–0.831)
2018	Bai et al. (94)	Meta-analysis	0.87 (0.0280)	0.87 (0.0248)
2020	Pala et al. (92)	Prospective, observational	0.668 (0.589–0.747)	0.722 (0.645–0.799)

Abbreviations; AUC = Area under the curve, BNP = Brain natriuretic peptide, CI = confidence interval, NT-proBNP = *n*-terminal pro brain natriuretic peptide, SE = Standard error.

Alternatively, NT-proBNP through its longer half-life, higher measured values, and lower intra-assay variability[89], could claim superiority as this may increase the chances of detecting a rise in plasma levels after a transient episode of AF. However, the current evidence directly comparing the performance of BNP and NT-proBNP for AF has focused on the ability of the peptides to distinguish CE stroke types from other stroke subtypes, and thus, only infer a possible role in diagnosing AF. To date, these studies have demonstrated mixed results, with some metanalysis including only studies measuring one of the biomarkers in heterogenous populations[90], [91], [92]. Moreover, Wachter et al.[93], in a direct comparison of both biomarkers, demonstrated an advantage of BNP over NT-proBNP in paroxysmal AF diagnosis. Meanwhile, Pala et al.[92], in another direct comparison of BNP and NT-proBNP for the diagnosis of AF, also demonstrated an advantage of BNP over NT-proBNP. However, BNP on occasion has demonstrated superior sensitivity, while NT-proBNP demonstrated superior specificity [92], [94]. Regardless, future larger studies in homogenous general populations are required to tease out the superior biomarker, or the individual roles each biomarker could play in AF prediction scenarios.

## 7 NPs and their potential role in ACM identification and management

As previously alluded to, the temporal dissociation of AF, amongst others, has blurred the anticoagulation rules for patients in AF. This has led to a reassessment of the atrial cardiomyopathy (ACM) theory and its link potential link to embolic events. Currently, no universal clinical ACM exists, but clinical definitions to date have encompassed ≥ 1 biomarkers of left atrial dysfunction, including left atrial enlargement on echocardiography, P-wave terminal force in lead V1 on the ECG, atrial fibrosis demonstrated by cardiac Magnetic Resonance Imaging, or elevation in serum biomarkers such as NT-proBNP, and left atrial diameter index[95], [96], [97]. Therefore, we can see a great heterogeneity of ACM definitions. Further work is required to understand the most suitable parameters to include in an ACM diagnosis, but also to define the magnitude of the TE risk in these patients.

It will be interesting to see how important a role NPs can play in the diagnosis of an ACM and prevention of TE events, given their potential ability to reflect a dysfunctional atrial environment. Given the discrepancy of ANP levels with AF chronicity and structural changes, its likely NTpro-BNP or BNP may be more reliable in identifying ACM[52]. However, given CNPs link with suppression of cardiac fibroblast proliferation, collagen synthesis and myocardial fibrosis, its future role in identifying and grading the degree of ACM cannot be understated[53]. This former concept is currently being investigated in the ongoing ARCADIA trial[98], where apixaban is being compared to aspirin for the prevention of recurrent stroke in those identified with an ACM (at least one of the following; P-wave terminal force > 5000 mV ms in ECG lead V1, serum NT-proBNP > 250 pg/mL or left atrial diameter index 3 cm/m^2^). Already a reduction of cardiovascular events, particularly ischemic stroke, in sinus rhythm patients taking a combination of an anticoagulant and aspirin over aspirin alone has been demonstrated in the COMPASS trial[99]. A similar result was seen in the Pegasus TIMI trial[100] where ticagrelor plus aspirin in patients in sinus rhythm with prior MI reduced ischemic stroke. However, the risk of major bleeding was also increased in the aforementioned study. Therefore, NT-proBNP, if confirmed as a biomarker of ACM, could further aid in the stratification of patients at risk of cardiovascular events. This would help further refine those who would benefit most from therapy, and further rationalize the assessment of bleeding risk.

## 8 Conclusion

In conclusion, NPs have shown promise in predicting AF. Further studies are required to confirm these results, and to potentially define the optimal NP subtypes, optimal cut off values in different clinical settings, and the frequency of checks required for AF screening. It is imperative that the ACM concept is further investigated, and its potential association with TE be clearly defined. If this association(s) can be confirmed and refined, these studies could and should strive to define the degree of benefit of these approaches on patient health outcomes, but also on health care economics. This could lead to more efficient AF screening, help risk stratify AF/ACM patients, and potentially reduce the burden of unnecessary screening and cost on healthcare systems, while reducing the clinical impact of these atrial pathologies.

## Declaration of Competing Interest

The authors declare that they have no known competing financial interests or personal relationships that could have appeared to influence the work reported in this paper.
